# Surface electromyography of the vastus lateralis and gluteus medius muscles in post-operative T3–L3 hemilaminectomy dogs: a prospective controlled observational study

**DOI:** 10.3389/fvets.2024.1431843

**Published:** 2024-08-01

**Authors:** John A. Schwartz, Sheila Carrera-Justiz, Jennifer A. Repac

**Affiliations:** ^1^Department of Comparative, Diagnostic & Population Medicine, College of Veterinary Medicine, University of Florida, Gainesville, FL, United States; ^2^Department of Small Animal Clinical Science, College of Veterinary Medicine, University of Florida, Gainesville, FL, United States

**Keywords:** rehabilitation, physical therapy, sports medicine, neurology, spinal cord injury

## Abstract

**Objective:**

The objective of this study was to determine if surface electromyography (sEMG) demonstrates differences in muscle activation between normal and dogs recovering from spinal cord injury due to intervertebral disk extrusion.

**Animals:**

Two groups of client-owned small-breed chondrodysplastic-type dogs were tested. Group 1 consisted of seven ambulatory paraparetic dogs that had undergone a hemilaminectomy procedure in the T3-L3 region for intervertebral disk extrusion 1 month prior. Group 2 was made up of seven normal dogs that had no history of intervertebral disk disease or spinal surgery.

**Procedures:**

Each subject walked 10 feet on a nonslip surface for at least five gait cycles for the sEMG to capture muscle activation of the vastus lateralis and gluteus medius, bilaterally. Muscle activation was quantified as the total myoelectric output area under the curve, averaged across all gait cycles.

**Results:**

Muscle activation was significantly greater in the post-operative hemilaminectomy group (*p* = 0.012). There was a significant difference in muscle activation between each hindlimb in the post-operative hemilaminectomy group, but not in the normal group. The muscle activation was significantly lower on the side that underwent surgery compared to the opposite limb (*p* = 0.0034).

**Conclusion and clinical importance:**

Post-operative hemilaminectomy dogs have greater hindlimb muscle activation compared to normal dogs, which likely represents a lack of descending inhibition secondary to upper motor neuron syndrome. The side of surgery is correlated with decreased muscle activation. Surface EMG can be used to evaluate muscle activity in dogs recovering from spinal decompression surgery.

## Introduction

Intervertebral disk disease (IVDD) is the most common spinal disorder and the leading cause of acute paralysis in dogs (1). Chondrodystrophic breeds are overrepresented, with Miniature and Standard Dachshunds most affected (2, 3). Currently, paresis is mainly evaluated through the use of observational gait scoring systems.

**Figure 1:**
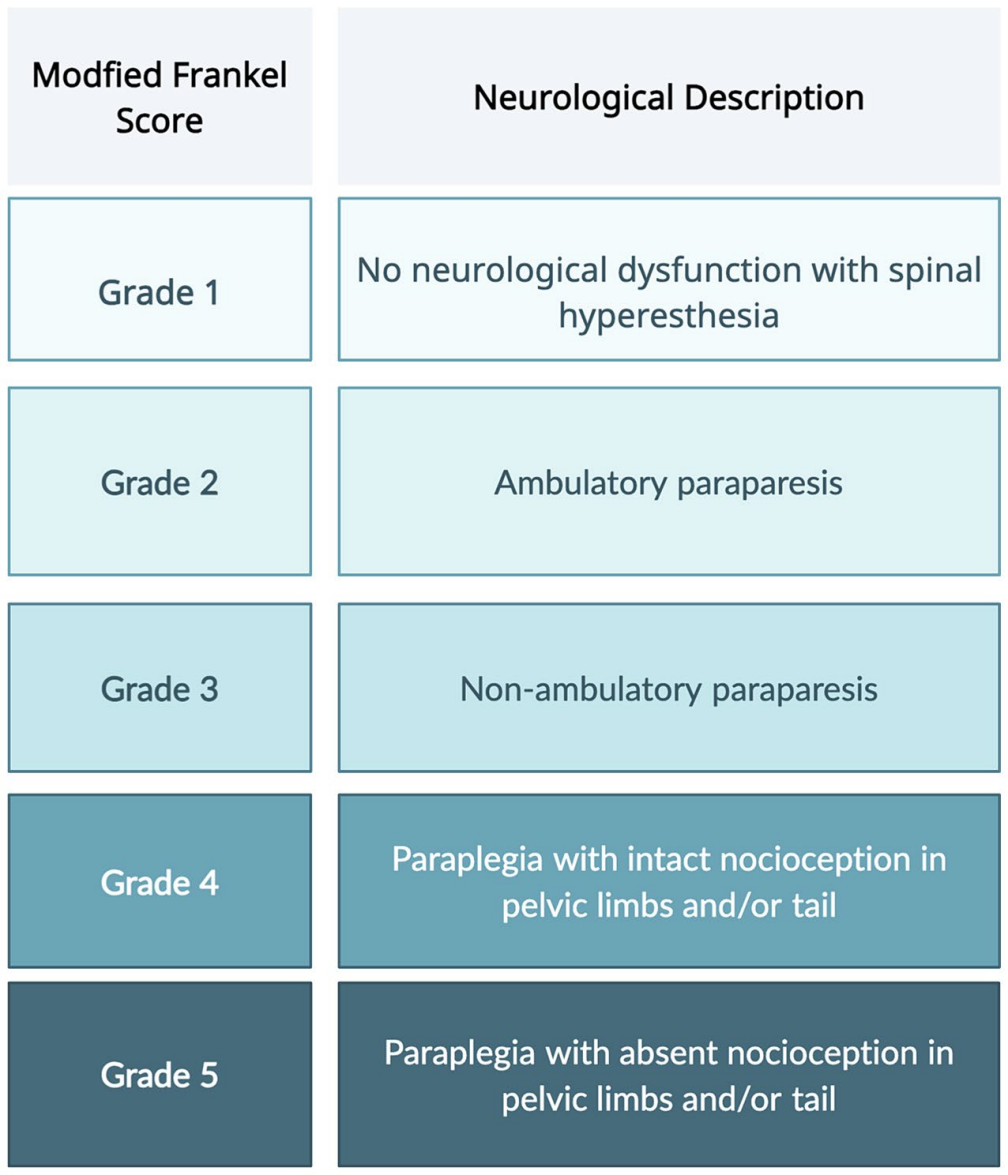
Modified Frankel score, used to described neurologic dysfunction.

The most commonly utilized neurologic scoring system, Modified Frankel Scoring (MFS), differentiates dogs into five categories based on the presence of motor, sensation, and ambulation status (Figure 1) (4–6). However, this scoring system fails to take into account the more subtle nuances of patient recovery. Dogs with a grade 2 or 3 MFS can have a wide range of motor function that is not conveyed by the scoring system. The American Spinal Injury Association (ASIA) impairment scale (AIS) is used in humans to evaluate functional impairment due to spinal cord injury (SCI). The scale includes tests not feasible to conduct in dogs because they require voluntary movement and verbal responses to stimuli (7).

Surface electromyography (sEMG) is a non-invasive technology that is used to measure muscle activity and myoelectric output. It offers an objective measure of neuromuscular function. Previous studies have used surface sEMG to measure muscle activity in normal dogs and dogs with orthopedic conditions such as hip osteoarthritis and cranial cruciate ligament rupture (8–10). Thus far, sEMG has not been used to evaluate neurological canine patients.

Surface EMG is well-established inhuman literature as a technology used in clinical rehabilitation, especially in people with spinal cord injuries (11). The only research that has evaluated muscle activity in dogs with myelopathies has used more invasive needle EMG technology in anesthetized patients (12, 13). No research has been performed using sEMG to measure muscle activity in dogs with myelopathies following hemilaminectomy. The objective of this study was to determine if sEMG demonstrates differences in muscle activation between normal and dogs recovering from intervertebral disk extrusion (IVDE). We hypothesize that the muscle activation pattern will be different in dogs 1-month post-operative thoracolumbar hemilaminectomy compared to normal dogs during walking.

## Materials and methods

Two groups of client-owned small-breed chondrodysplastic-type dogs weighing up to 20 kilograms were tested (Table 1). Group 1 was comprised of seven ambulatory paraparetic dogs that had a history of intervertebral disk extrusion with a hemilaminectomy procedure in the T3-L3 region that was performed 1 month prior. Group 2 was made up of seven dogs that had no history of intervertebral disk disease or spinal surgery with normal neurological and orthopedic examinations. The inclusion and exclusion criteria of each respective group was as follows:

**Table 1:** Demographic data; group 1 (control group) and group 2 (post-op hemilaminectomy).

	Patient	Breed	Age (years)	Sex	Weight (kg)	Surgery	MFS grade
Group 1							
	1	Miniature Dachshund	8	M	7.6	n/a	n/a
	2	Miniature Dachshund	3	F	4.2	n/a	n/a
	3	Chihuahua Mix	4	M	4.8	n/a	n/a
	4	French Bulldog	1	M	15.7	n/a	n/a
	5	Miniature Dachshund	8	M	7.6	n/a	n/a
	6	Chihuahua Mix	5	F	6.8	n/a	n/a
	7	Miniature Dachshund	8	M	7.3	n/a	n/a
Group 2							
	8	Chihuahua	10	M	6.1	Right T11-T12	2
	9	Havanese mix	3	F	6.2	Right T11-T12	2
	10	Miniature Dachshund	11	M	5.0	Left L1-L2	2
	11	French Bulldog	5	M	18.2	Right T13-L1	2
	12	Papillon	8	M	4.7	Right T13-L1	2
	13	Chihuahua Mix	6	M	16.0	Right T11-T12	2
	14	Miniature Dachshund	5	F	4.9	Right T11-T12	2

Group 1 (Post-hemilaminectomy, *n* = 7) inclusion criteria:

- Ambulatory paraparesis
- Chondrodysplastic breeds
- Weighing up to 20 kg
- Body condition score of 5/9 and muscle condition score of 3/3
- Dogs with a history of intervertebral disk extrusion with history of hemilaminectomy in the T3-L3 region, performed 4–6 weeks prior.
- Normal orthopedic exam
- Dogs with no other evidence of disk extrusion in other spinal segments.

Group 2 (Control dogs, *n* = 7) inclusion criteria:

- Chondrodysplastic breeds
- Weighing up to 20 kg
- Body condition score of 5/9 and muscle condition score of 3/3
- Normal neurological and orthopedic exam
- No history of IVDD or spinal surgery

Exclusion criteria for both groups:

- Orthopedic abnormalities
- Other concurrent spinal cord or neurologic disease
- Dermatologic conditions that interfere with sensor placement
- Temperament not conducive to sensor placement
- Serious comorbidities or medication (e.g., anti-epileptic drugs, glucocorticoids) that may affect mobility

**Figure 2:**
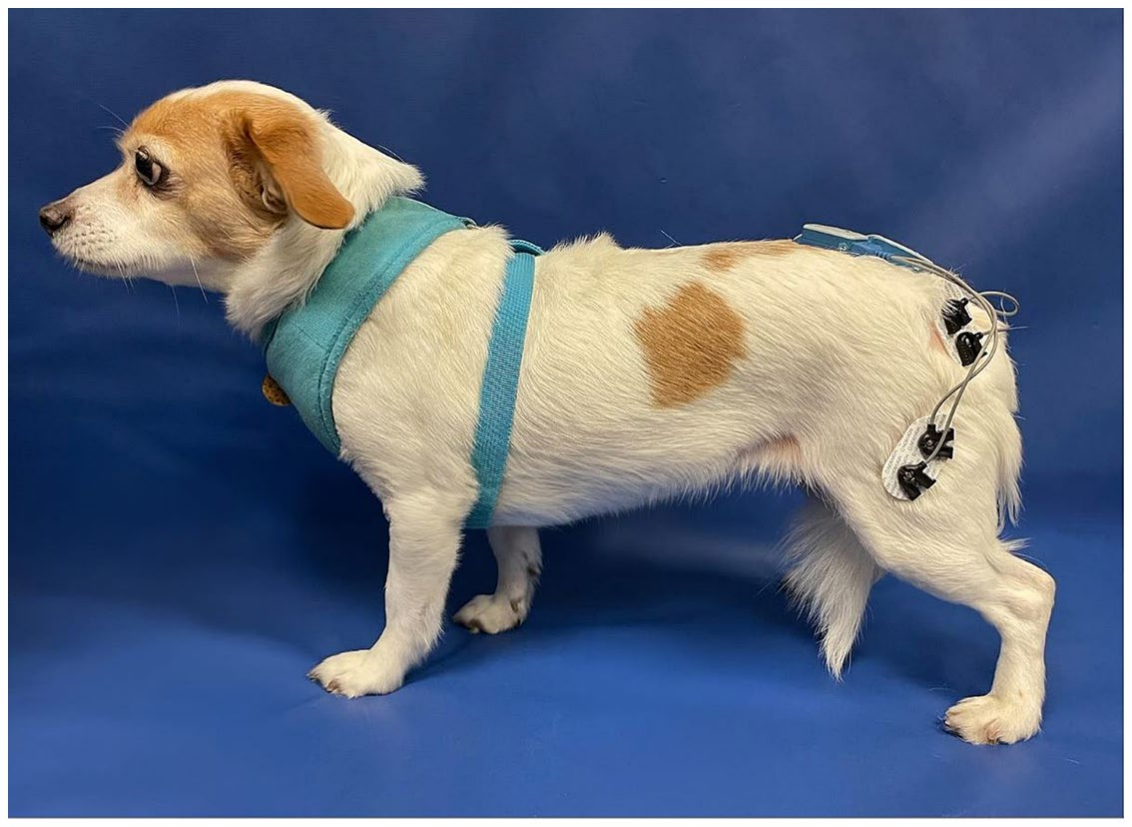
The position of the dual electrodes and sensors over the left vastus lateralis and gluteus medius muscles.

All animals were assessed by a board-certified Neurologist or neurology resident for a neurological examination and a board-certified Sports Medicine and Rehabilitation specialist for an orthopedic examination prior to study enrolment. During the same visit, a telemetric unit (Myomotion; Noraxon United States, Inc., Scottsdale, AZ) was used to measure muscle activity. The disposable self-adhesive dual electrodes with low impedance solid gel (2 cm spacing) and sensors were applied and positioned using adhesive barrier wipes (Skin-Tac™; Torbot Group Inc., Toledo, OH) to clean and shaven skin over the vastus lateralis and gluteus medius muscles, bilaterally as previously described (Figure 2) (9). Each subject was leash-walked with a slip lead or encouraged to walk toward an investigator at a comfortable walking pace over a marked 10 ft. nonslip surface for the sEMG to capture muscle activation over five gait cycles within the defined distance. The data were processed and smoothed to remove noise using an infinite impulse response (IIR) bidirectional lowpass Butterworth filter (<50 Hz and > 450 Hz) (14–16). Data were normalized to the peak amplitude within each animal’s gait cycle using a 500 ms window. The total sEMG burst area was calculated by adding up all points within the burst after subtracting the background sEMG and then averaged across all gait cycles to create a mean area under the curve as a percentage of muscle activation.

### Statistics

Statistical analysis was performed using JMP®, Version 17 (SAS Institute Inc., Cary, NC). A linear mixed model was used to analyze the data with the patient as a random effect and surgical side, muscle, and their interaction as fixed effects. Model assumptions, normality, and constant variance were checked via inspection of model residuals. Tukey’s multiple comparison procedure was used to test for pairwise mean comparisons. A linear contrast was used to test for overall differences between surgical groups. A *p*-value < 0.05 was considered statistically significant. Based on previous studies, we predicted mean muscle activation would have a standard deviation of 0.95 and the correlation between normal and post-operative dogs would be 0.5 (8). Based on our parameter choices, we used 14 participants for a desired power of 0.90 and a Type I error rate of 0.05.

## Results

**Figure 3:**
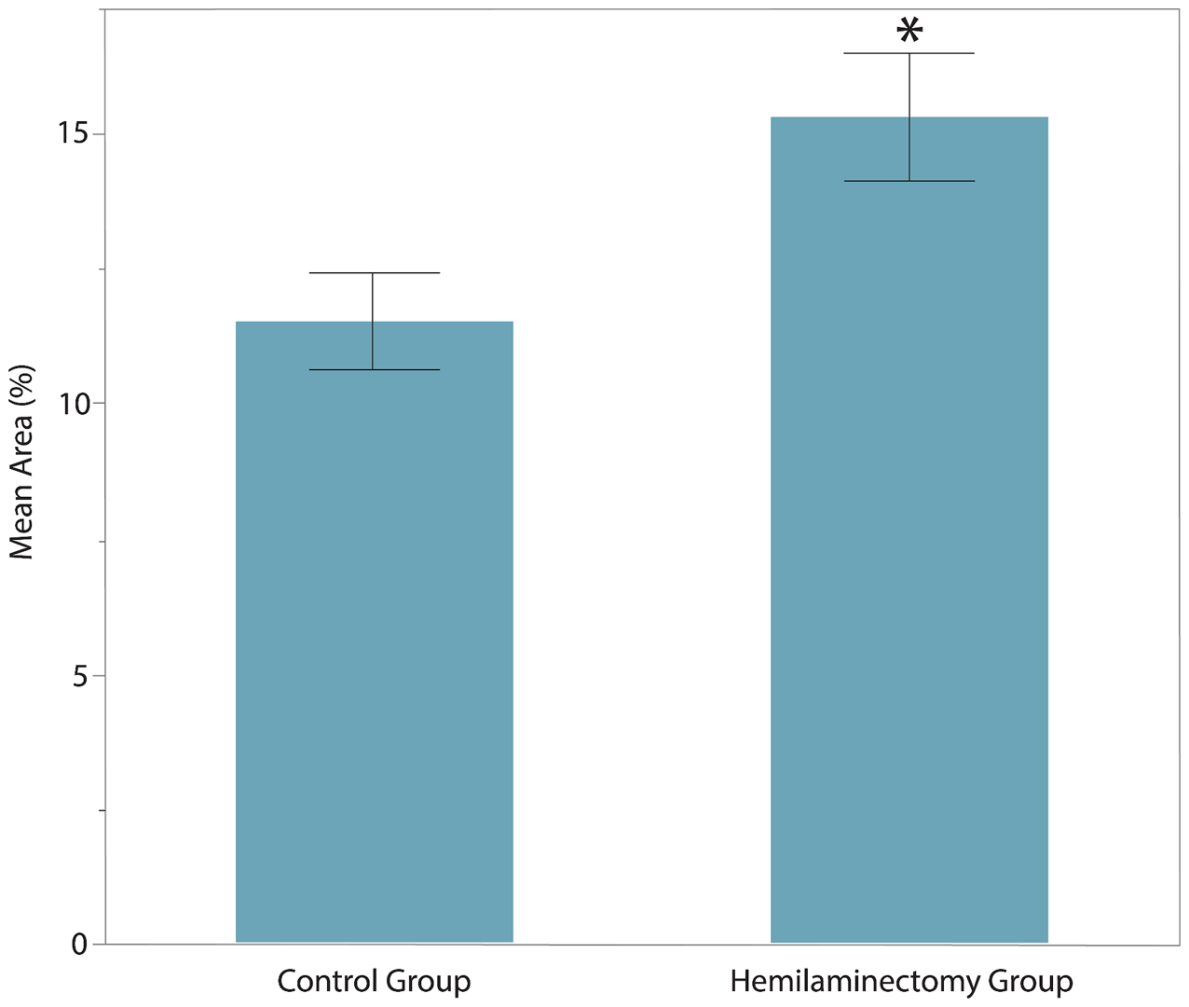
Muscle activity of vastus lateralis and gluteus medius between control group and post-operative hemilaminectomy group. The hemilaminectomy group had significantly greater muscle activity (15.34 ± 2.41%) than the control group (11.52 ± 2.43%, *represents significance at *p* = 0.012).

**Figure 4:**
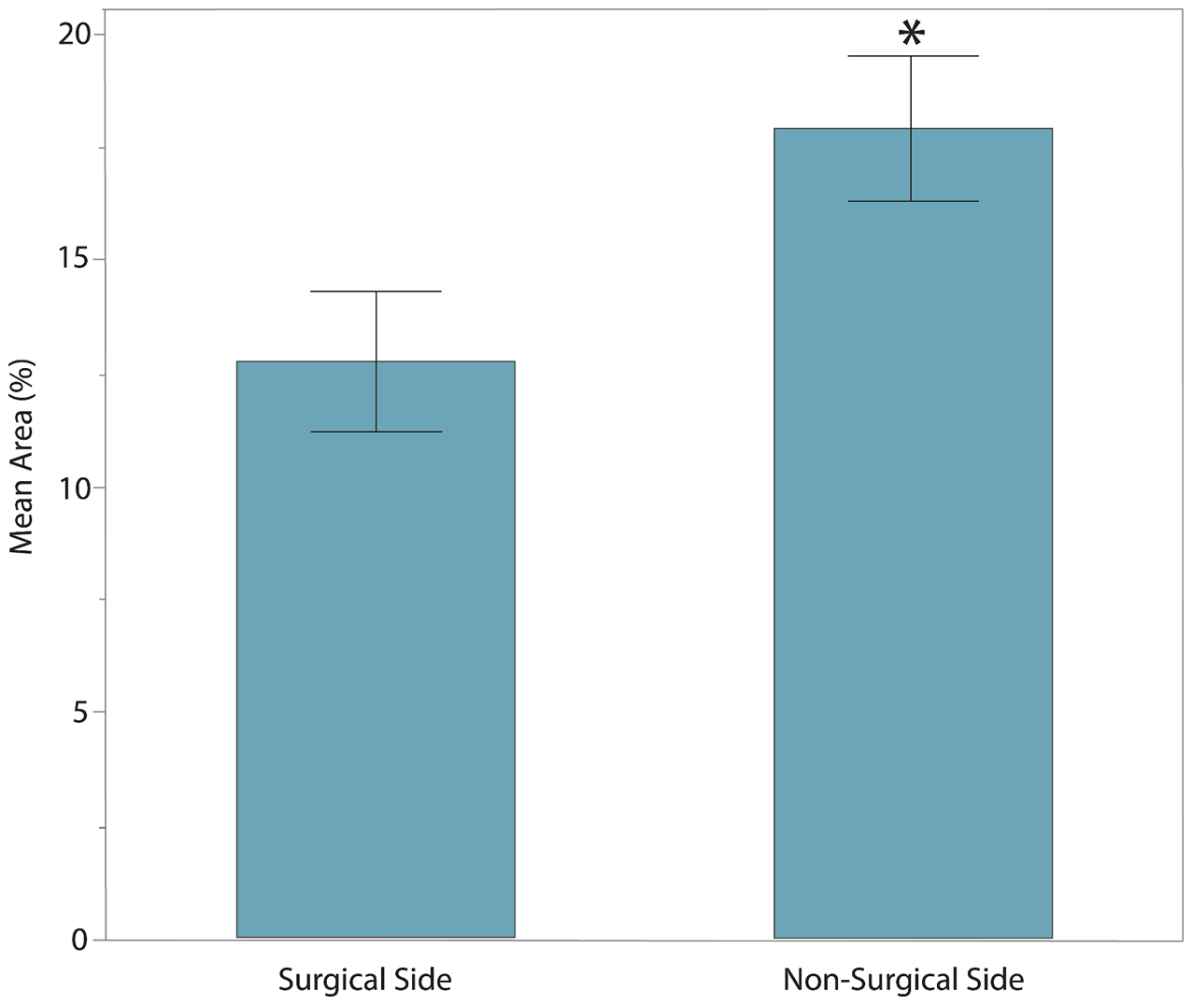
The sum of combined muscle activity between each hindlimb within the post-operative hemilaminectomy group. The muscle activity was significantly greater in the limb on the non-surgical side (17.92 ± 4.45%) compared to the post-hemilaminectomy group (12.77 ± 4.67%, *represents significance at *p* = 0.0034).

The age, sex, MFS, surgical location, and body weight of all dogs of the post-hemilaminectomy dogs are described in Table 1. There were no statistically significant differences between the two groups with respect to age, sex, and weight (*p* > 0.05). Muscle activity was significantly greater in the hemilaminectomy group (*p* = 0.012) compared to the normal group (Figure 3; Table 2). A significant difference was also seen between each hindlimb within the post-operative group. The sum of the combined muscle activity was significantly greater in the contralateral limb opposite the limb on the surgical (*p* = 0.0034; Figure 4). There were no significant differences in muscle activity between the different muscle groups.

**Table 2:** Mean area and standard deviation of muscle activation; group 1 (control group) and group 2 (post-op hemilaminectomy).

	Patient	Total Mean ± SD	Left Hindlimb Mean	Right Hindlimb Mean
Group 1				
	1	9.45 ± 3.88%	12.25 ± 2.05	6.65 ± 3.10
	2	9.46 ± 8.36%	8.15 ± 8.00	10.77 ± 11.79
	3	11.65 ± 2.67%	12.48 ± 3.85	10.83 ± 1.94
	4	11.55 ± 1.80%	11.40 ± 2.69	11.70 ± 1.60
	5	10.33 ± 0.29%	10.55 ± 0.21	10.10 ± 0
	6	16.55 ± 5.27%	15.25 ± 0.92	17.85 ± 8.70
	7	11.66 ± 6.36%	12.00 ± 1.70	11.32 ± 10.87
Group 2	Patient	Total Mean ± SD	Surgical Side Mean	Non-Surgical Side Mean
	8	13.94 ± 12.56%	3.08 ± 0.76%	24.80 ± 01.13%
	9	12.16 ± 2.17%	11.11 ± 1.82%	13.20 ± 2.55%
	10	13.65 ± 2.28%	13.60 ± 3.68%	13.70 ± 1.14%
	11	17.13 ± 5.09%	15.10 ± 7.21%	19.15 ± 3.04%
	12	18.26 ± 5.92%	14.62 ± 7.19%	21.90 ± 0.42%
	13	17.98 ± 7.71%	17.45 ± 3.46%	18.52 ± 12.82%
	14	14.30 ± 4.69%	14.40 ± 5.37%	14.19 ± 6.10%

## Discussion

The use of sEMG is a widely accepted outcome measure in both human spinal cord injury research and clinical physical rehabilitation (17). In veterinary literature, previous studies have been primarily focused on using sEMG to measure muscle activity in normal dogs and in dogs with orthopedic conditions, but never those affected by neurologic disease.

This study found significantly greater muscle activation in the post-op hemilaminectomy compared to the control group. Thus, our hypothesis that the muscle activation pattern will be different in dogs one-month post-operative thoracolumbar hemilaminectomy compared to normal dogs during walking was supported. However, we initially expected that the post-op hemilaminectomy group would have decreased muscle activity due to paresis.

Dogs with intervertebral disk extrusions (IVDE) in the T3-L3 spinal cord segment typically display signs of upper motor neuron (UMN) syndrome. This consists of paresis, ataxia, and spasticity in the hindlimbs. The UMN pathways inhibit both the extensor and flexor muscles. However, most T3-L3 lesions that affect these pathways release the extensor motor neurons from inhibition, resulting in hypertonia (18). The increase in muscle activity can cause paresis and ataxia because the normal inhibition of these muscles is what results in a smooth and regulated gait. The greater muscle activity in the post-op hemilaminectomy group may be explained by this hypertonia.

Within the post-op hemilaminectomy group, there was also significantly less muscle activation in the limb on the surgical side compared to the non-surgical side. The laterality of the surgical decompression is determined based on the side with the greatest spinal cord compression. For this reason, dogs are usually more paretic on the same side as the surgical site. That the increased muscle activity of the limb on the non-surgical side is due to a combination of increased UMN spasticity and increased voluntary motor function. Human medical literature shows that patients with SCI can experience increased muscle activity due to spasticity. For example, in studies using sEMG, involuntary muscle activity at rest was found to be significantly higher in SCI participants compared to able-bodied control participants (17). Additionally, using sEMG has been shown to measure reflex hyperexcitability and determine the occurrence of muscle spasms in individuals with SCI (19, 20).

This study had limitations due to the sample size and the patient population we used. Although a power analysis was performed, the sample size remained relatively limited. It was also challenging to fit sEMG sensors and electrodes on the hindlimbs of chondrodysplastic dogs weighing less than 20 kg. This is why sensors were placed on only two muscles (vastus lateralis and gluteus medius muscles), compared to previous studies, which also include the biceps femoris. Previous studies have typically used larger breed dogs, such as Labrador Retrievers, Golden Retrievers, Weimaraners, and shepherds (8, 9, 14, 21). Chondrodystrophic dogs were selected for this study because they are most representative of the population undergoing hemilaminectomies. In addition to smaller anatomy, variations in body condition and skin movement can make it difficult to isolate individual muscle activity (22, 23). In humans, it has been shown that increased body fat and body mass index can contribute to a lower recording of bioelectrical activity when using sEMG (24). Surface EMG cannot measure the activity of deep muscles so it is not as precise as conventional EMG, which uses a needle that can be placed into the exact muscle body of interest. This is why all the patients included in this study had to have an ideal body condition score of 5/9 and a normal muscle condition score of 3/3.

In human sEMG studies, data is normalized to a maximum voluntary contraction, which helps define muscle activity as a percentage of that maximum value (14). This cannot be performed in dogs, so a maximum dynamic contraction is used instead as a baseline by having a dog perform a high-intensity exercise (25). Because our patient population was recovering from hemilaminectomy surgery and still had neurological deficits, a maximum dynamic contraction could not be used to normalize the data. Instead, we measured the muscle burst duration as an area under the curve as a percent during the gait cycle to obtain the muscle activity values (26).

While sEMG is non-invasive and relatively user-friendly, EMG overall has several limitations. A significant drawback is the requirement for isometric contractions to establish a reliable quantitative relationship between EMG signals and muscle force (27). Non-linearities and signal non-stationarities are introduced by anisometric contractions, which can complicate analysis and interpretation. Additionally, intrinsic anatomical and physiological factors, such as the number of active motor units, their proximity to the electrode, the detection volume of the electrode, and the presence of subcutaneous tissue, can influence the EMG signal’s amplitude. The interpretation of the EMG signal is further complicated by crosstalk from nearby muscles and the instability of motor unit activation patterns during dynamic contractions (27).

Future studies could potentially use scales such as the Ashworth or Olby scales to better understand the relationship between muscle activity and clinical status of disease than the more simplified MFS that was used in this study. The Modified Ashworth Scale is the predominant clinical measure utilized to evaluate muscle spasticity in human patients diagnosed with neurological conditions. The scale assesses resistance experienced during passive range of motion and is used to evaluate the efficacy of pharmacologic and physical rehabilitation interventions (28). The Olby scale is a 14-point SCI grading scale that was validated in dogs to assess neurological function following spinal cord injury (29). It evaluates voluntary movement, muscle tone, and sensation to accurately quantify the extent of recovery in dogs following SCI.

Compared to human medicine, there is less research and validation of sEMG techniques in veterinary medicine (30). This is mainly due to the lack of standardized protocols, reference values, and established clinical applications. Widely available sEMG systems are also typically designed for human use, which can make it difficult for veterinary-specific applications. Though additional research is needed, this study has shown that sEMG can be used to measure muscle activity in ambulatory paraparetic dogs following SCI.

## Conclusion

Surface EMG can be used to evaluate muscle activity in dogs recovering from T3-L3 IVDE. There is greater muscle activity in dogs that have undergone hemilaminectomy surgery compared to normal dogs. This increase is most likely due to hypertonia resulting from UMN syndrome. To gage the recovery progress of dogs who have undergone spinal decompression surgery, research is needed to determine whether sEMG can be employed as an effective prognostic indicator for dogs recovering from a T3-L3 myelopathy due to IVDE.

## Data availability statement

The raw data supporting the conclusions of this article will be made available by the authors, without undue reservation.

## Ethics statement

The animal studies were approved by University of Florida’s Institutional Animal Care & Use Committee and Veterinary Hospitals Research Review Committee. The studies were conducted in accordance with the local legislation and institutional requirements. Written informed consent was obtained from the owners for the participation of their animals in this study.

## Author contributions

JS: Data curation, Formal analysis, Funding acquisition, Investigation, Software, Validation, Visualization, Writing – original draft, Writing – review & editing. SC-J: Supervision, Writing – review & editing, Methodology. JR: Conceptualization, Investigation, Methodology, Project administration, Resources, Supervision, Writing – review & editing, Visualization.
